# Age-related self-overestimation of step-over ability in healthy older adults and its relationship to fall risk

**DOI:** 10.1186/1471-2318-13-44

**Published:** 2013-05-07

**Authors:** Ryota Sakurai, Yoshinori Fujiwara, Masami Ishihara, Takahiro Higuchi, Hayato Uchida, Kuniyasu Imanaka

**Affiliations:** 1 Health Promotion Science, Tokyo Metropolitan University, 1-1 Minami-Osawa, Hachioji-shi, Tokyo 192-0397, Japan; 2 Research Fellow of the Japan Society for the Promotion of Science, Japan Society for the Promotion of Science, 5-3-1 Kojimachi, Chiyoda-ku, Tokyo 102-0083, Japan; 3 Research Team for Social Participation and Community Health, Tokyo Metropolitan Institute of Gerontology, 35-2 Sakae-cho, Itabashi-ku, Tokyo, 173-0015, Japan; 4 School of Human Science & Environment, University of Hyogo, 8-2-1, Gakuennishi-machi, Nishi-ku, Kobe-shi, Hyogo, 651-2197, Japan

**Keywords:** Aging, Self-assessment, Stepping-over, Accidental falls, Judgment, Safety, Psychomotor performance

## Abstract

**Background:**

Older adults could not safely step over an obstacle unless they correctly estimated their physical ability to be capable of a successful step over action. Thus, incorrect estimation (overestimation) of ability to step over an obstacle could result in severe accident such as falls in older adults. We investigated whether older adults tended to overestimate step-over ability compared with young adults and whether such overestimation in stepping over obstacles was associated with falls.

**Methods:**

Three groups of adults, young-old (age, 60–74 years; n, 343), old-old (age, >74 years; n, 151), and young (age, 18–35 years; n, 71), performed our original step-over test (SOT). In the SOT, participants observed a horizontal bar at a 7-m distance and estimated the maximum height (EH) that they could step over. After estimation, they performed real SOT trials to measure the actual maximum height (AH). We also identified participants who had experienced falls in the 1 year period before the study.

**Results:**

Thirty-nine young-old adults (11.4%) and 49 old-old adults (32.5%) failed to step over the bar at EH (overestimation), whereas all young adults succeeded (underestimation). There was a significant negative correlation between actual performance (AH) and self-estimation error (difference between EH and AH) in the older adults, indicating that older adults with lower AH (SOT ability) tended to overestimate actual ability (EH > AH) and vice versa. Furthermore, the percentage of participants who overestimated SOT ability in the fallers (28%) was almost double larger than that in the non-fallers (16%), with the fallers showing significantly lower SOT ability than the non-fallers.

**Conclusions:**

Older adults appear unaware of age-related physical decline and tended to overestimate step-over ability. Both age-related decline in step-over ability, and more importantly, overestimation or decreased underestimation of this ability may raise potential risk of falls.

## Background

Falls are a major concern in health care for older adults because most falls are associated with a high risk of fractures, resulting in a need for long-term care [1-3]. The etiology of falls is multifactorial and among them a most important problem leading to falls [4-7] would be a tripping when stepping over an obstacle. However, previous studies have primarily focused on age-related decline in physical ability or muscular strength [1,3,8-10] and visuomotor control of foot movements [11-14] as predominant factors in tripping during the step-over action. Although increasing evidence implicates cognitive factors, such as attention, executive function, and problem solving in tripping [3,15-17], whether age-related changes in self-estimation of physical ability in older adults is a crucial cause of tripping while stepping over an obstacle remains unclear.

Robinovitch and Cronin [18] highlighted a tendency for older adults to overestimate, and young adults to underestimate reaching ability. More importantly, overestimation of reaching ability in older adults was more evident for older adults whose reaching ability declined. This has recently been supported by Butler et al. [19], who have also demonstrated evidence for overestimation of reaching ability in older adults, although the overestimation of reaching ability was not significantly associated with the occurrence of falls within the year before and after the study.

No significant association between overestimation of reaching ability and occurrence of falls [19] might be because reaching ability affects postural balance [20] but may not be directly associated with other motor actions, such as locomotion and stepping over an obstacle (which could relate to possible falls) [9] in older adults. Alternatively, if older adults tend to overestimate step-over ability, as it is with reaching, this may increase the risk of tripping during the step-over action, which could lead to falls in older adults [3-7]. To date, however, no study has addressed these issues by using a step-over task.

In the present study, we therefore investigated (i) whether older adults tended to overestimate (or underestimate to a lesser extent than younger adults) step-over ability and, if so, (ii) whether such overestimation (or decreased underestimation) in stepping over obstacles was associated with falls. To this end, community-dwelling older adults were tested with our original step-over test (SOT). They were assigned to either young-old (60–74 years) or old-old (≥75 years) age groups. Younger adults (18–35 years) were also tested as a controls. The accuracy (i.e., overestimation and underestimation) of self-estimation of step-over ability in the SOT was evaluated in terms of the difference between self-estimated maximum height (EH) and actually performed maximum height (AH). We then examined whether the extent of over/underestimation was related to the occurrence of falls within the past year.

## Methods

### Participants

Community-dwelling healthy older adults (n, 567; mean age [SD], 72.2 [5.6] years; 80.6% were female) from urban and local areas were recruited via direct mail or newsletter. Seventy-three older adults were excluded on the basis of the following exclusion criteria: (i) severe conditions or injuries (e.g., stroke) in the 3 months preceding the study; (ii) uncorrected visual defects leading to inability to visually identify the experimental device (corrected binocular visual acuity < 1.0 identified by a visual acuity examination using the Landolt ring chart) [21]; (iii) neuromuscular/mental disorders or cognitive impairment (Mini-Mental State Examination [MMSE], <27) [22]; (iv) use of a walking aid such as a cane; and (v) addiction to psychoactive substances or tranquilizers. This yielded 494 older adults (mean age [SD], 72.1 [5.4] years; 79.8% were female; no existing instrumental activities of daily living [IADL] problems identified by the TMIG-Index of Competence [23]) participating at subsequent measurements. Seventy-one healthy young adults (mean age [SD], 22.0 [3.1] years; 49.3% were female) were recruited from the Tokyo Metropolitan University as controls. They had no physical, neurological, or mental disorders, and used no medication. There was a large difference in male–female ratio of participants between young and older groups. This was simply because the number of young females did not reach a similar number of female participants of older adults.

The participants were assigned to 3 age groups, that is, the young group (18–35 years; n, 71), young-old group (60–74 years; n, 343), and old-old group (≥75 years; n, 151). Written informed consent was obtained from each participant before participation in the study. The study was conducted in accordance with the ethical standards of the Declaration of Helsinki (1983), and the research protocol was approved by the Tokyo Metropolitan Institute of Gerontology.

### Interview and questionnaire items

During the first session of this study, all the participants were interviewed by either a physician or a physical therapist (author RS) to assess their health-related characteristics (e.g., demographics, anamnesis, history of hospitalization, and medication use) and individual history of falls within the previous year. Falls were defined as any unintentional drops/falls to the ground or floor, excluding bicycle accidents, accidental contact with furniture, walls, or other environmental structures and sudden cardiovascular or central nervous system events [24]. The participants in the young group were also interviewed, excluding fall history.

### Step-over test

**Figure 1:**
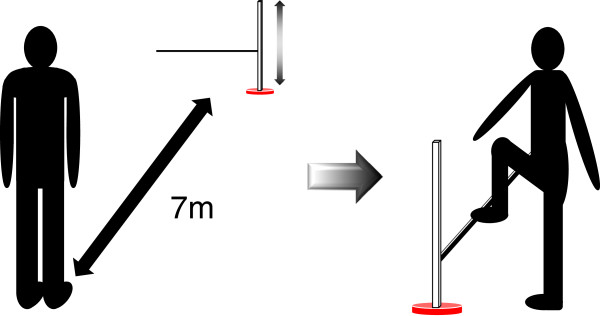
**Schematic illustration of the step-over test (SOT). **Participants observed a horizontal bar at a 7-m distance and estimated their maximum height (estimated height, EH) which they believed they could successfully step over. After the estimation, both the success rate of whether they stepped over the bar at EH and their actual height (AH) were measured.

During the second session, actual SOT performance and accuracy of self-estimation were measured using the original SOT. Participants first performed the self-estimation test and then the actual step-over task (Figure 1).

For the self-estimation test, a horizontal black wooden bar (25 × 25 × 900 mm) was placed 2 m in front of a white background wall. The participants were asked to observe it from a 7-m distance. The experimenter manually adjusted the height of the bar either from 10 cm to 80 cm (ascending direction) or from 80 cm to 10 cm (descending direction) slowly. While the bar was moving, the participants were asked to verbally respond “stop” at the point they believed the bar had reached the maximum height that they could step over. They were instructed to imagine stepping over the bar with the body facing straight ahead and with no restrictions to the posture except for jumping. The participants were allowed to amend their estimated maximum height after the verbal response and the experimenter manually adjusted the bar height accordingly. No time restriction was imposed on their estimation. Four trials of self-estimation were performed with 2 ascending and 2 descending series of manipulations of the bar height. The mean estimated maximum height (EH) of the 4 trials was calculated per participant.

Subsequently, the participants were asked to approach the bar to step over it with the bar set at the individual EH. If they failed to step over the bar at the EH (i.e., touching/kicking the bar with the foot/lower limb), the bar was lowered by 3 cm. Alternatively, if they succeeded at the EH, the bar was raised by 3 cm. They were then asked to step over the bar again at the new height. This was repeated until they either succeeded or failed in the step-over action, and the final height at which they were successful in 2 consecutive trials was recorded as the individual AH.

Because the ability to step over an obstacle generally correlates with lower limb length [25], the EH and AH were divided by the length (distance from the greater trochanter to the ground through the lateral malleolus) of the lower limb and these ratios were used as the individual representative EH and AH for subsequent analyses. The difference between EH and AH (Δ height) was then calculated to determine the accuracy, or bias error (i.e., underestimation or overestimation), of self-estimation of step-over ability. The Δ height was then standardized according to the AH with the following formula: [(EH - AH)/AH × 100]. We also calculated the percentage of participants who failed to step over the bar at the EH in each age group.

We confirmed high test-retest reliability (at intervals of a month) for EH and AH in 40 young and 40 older adults (each intraclass correlation coefficient > 0.9).

### Design and statistical analysis

To examine age-related differences in measurement variables among the 3 age groups (i.e., young, young-old, and old-old groups), chi-square tests were performed on both the percentage of participants who failed to step over the bar at the EH and the percentage of female participants (sex ratio). For the variables of age and leg length (LL), a multivariate analysis of variance (MANOVA) was performed. For the SOT performance (EH and AH), a repeated measures analysis of variance (ANOVA) adjusted by sex was performed for the 3 age groups. The relationships between the self-estimation error (Δ height) and respective variables of age, EH, and AH were examined with correlation analyses as well. The self-estimation error (Δ height) was compared for fallers and non-fallers with a repeated measures ANOVA adjusted by sex. The percentage of participants who failed to step over the bar at the EH (i.e., who showed overestimation) was also compared for fallers and non-fallers with a chi-square test. All the statistical analyses were performed with the PC-compatible version of IBM SPSS Statistics Version 19.0 (SPSS Inc., Chicago, IL).

## Results

### Demographic and anthropometric characteristics

For % female, age, and leg length in Table 1, a MANOVA and post hoc analyses revealed that both the young-old and old-old groups showed significantly larger number of females (sex, % female), much older age, and shorter leg length than those of the young group (*p* < 0.016 for all with Bonferroni correction).

**Table 1:** Characteristics of 3 groups for sex, age, leg length, and the number of participants with overestimation in SOT

	**Young adults**	**Young-old adults**	**Old-old adults**	**p value**
	**(n=71)**	**(n=343)**	**(n=151)**	
Sex, % female	49.3	83.1	72.2	<0.01
Age	22.0 ± 3.1	69.4 ± 3.3	78.5 ± 3.5	<0.01
Leg length, cm	80.9 ± 5.3	74.7 ± 4.4	73.8 ± 5.2	<0.01
Δ height > 0, n(%)^*1^	0	39 (11.4)	49 (32.5)	<0.01

MANOVA was adjusted for sex in age and leg length.

^※1^, Percentage of participants who failed to step over the bar at estimated height (EH) (i.e., overestimation). A chi-square test was performed to compare the 2 older adult groups alone because all the young adults succeeded.

### Step-over ability and its self-estimation

As shown in Table 1 (Δ height > 0), all the young adults successfully stepped over the bar at EH, whereas 11.4% of young-old adults and 32.5% of old-old adults failed to step over the bar at EH. A chi-square test on these data showed that the percentage of the old-old adults was significantly larger than that of the young-old adults (*p* < 0.01), with no significant sex differences for these data.

**Figure 2:**
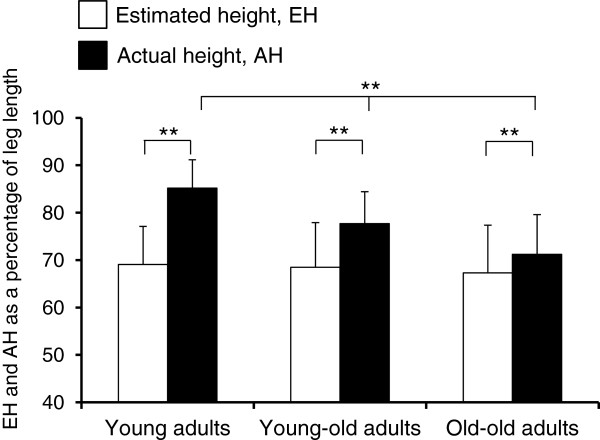
**EH and AH of the step-over test (SOT) for the 3 age groups. **The main effects of SOT performance (F_1, 562 = _34.9, *p *< 0.01) and age (F_2, 562 = _34.6, *p *< 0.01) were significant, with the interaction between the two factors being also significant (F_2, 562 = _40.2, *p* < 0.01). The graphical symbol of “**” indicates *p *< 0.01. EH, estimated height; AH, actual height.

Figure 2 shows SOT performance (EH and AH) for the 3 age groups. Mixed-design two-way ANOVA showed that actual SOT ability (AH) significantly decreased as age increased (*p* < 0.01 for all pairs of 3 groups), whereas self-estimation (EH) of SOT ability appeared to remain unchanged as age increased.

**Figure 3:**
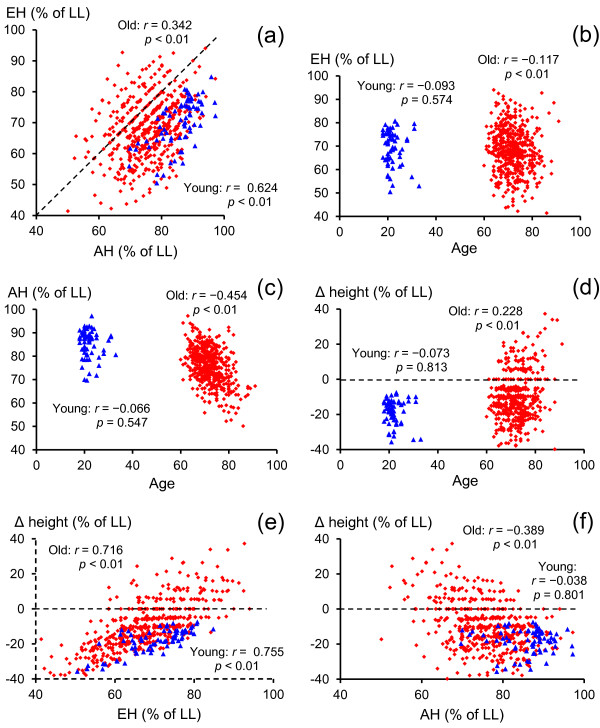
**Scatter diagrams of the step-over test (SOT) variables and age. **Panels display; (**a**), the scatter diagram of EH and AH; (**b**), the scatter diagram of EH and age; (**c**), the scatter diagram of AH and age; (**d**), the scatter diagram of Δ height and age; (**e**), the scatter diagram of Δ height and EH; (**f**), the scatter diagram of Δ height and AH. The filled squares represent the elderly participants, whereas the filled triangles represent the young participants. The positive Δ height values represent overestimations, that is, situations in which the participants were unable to step over the bar positioned at the estimated height (**d**, **e**, and **f**). EH, estimated height; AH, actual height; LL, leg length.

Figure 3 shows scatter plots depicting the relationships among EH, AH, Δ height, and age. In Figure 3a, the young adults show a high correlation coefficient between EH and AH (r = 0.624, *p* < 0.01), whereas the older adults show a relatively low correlation coefficient (r = 0.342, *p* < 0.01), which was significantly smaller than that for the young adults (*p* < 0.01). This indicated that the self-estimation of the older adults was inaccurate compared with the young adults (coefficient of determination; R^2^ = 0.389 vs. 0.116).

As shown in Figures 3b and 3c, the correlation coefficients between EH and age (r = -0.117, *p* < 0.01) and between AH and age (r = -0.454, *p* < 0.01) were significant for the older adults, with the AH–age correlation coefficient significantly larger than the EH–age correlation (*p* < 0.01). This was not the case for the young adults (r = -0.093 and -0.066).

Figure 3d shows that the self-estimation error (Δ height) was significantly correlated with age for the older adults (r = 0.228, *p* < 0.01) but not for the young adults (r = -0.073, *p* > 0.1). As shown in Figure 3e, the Δ height was significantly positively correlated with EH for both the older (r = 0.716, *p* < 0.01) and young (r = 0.755, *p* < 0.01) adults, indicating that self-estimation error generally reflect EH for both young and older adults. In contrast, shown in Figure 3f, the Δ height was significantly negatively correlated with AH in the older adults (r = -0.389, *p* < 0.01), with no significant correlation in the young adults (r = -0.038, *p* > 0.1). This indicated that the self-estimation error in the older adults significantly increased (decreasing underestimation and approaching overestimation) as the physical step-over ability deteriorated.

### Self-estimation of step-over ability and falls

**Figure 4:**
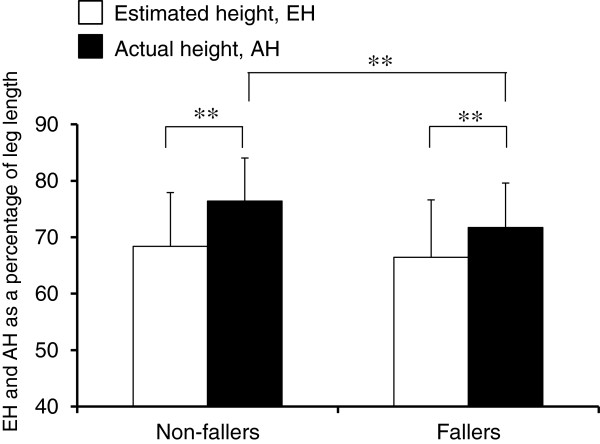
**Comparisons of SOT performance of EH and AH between the fallers and non-fallers.** The main effects of SOT performance (F_1, 492 = _9.35, *p *< 0.01) and non-fallers/fallers (F_1, 492 = _13.2, *p *< 0.01) were significant, with the interaction between the two factors being also significant (F_1, 492 = _4.58, *p *< 0.05). The graphical symbol of “**” indicates *p *< 0.01. SOT, step-over test; EH, estimated height; AH, actual height.

Interviews for falls revealed that 40 (11.6%) young-old and 32 (21.2%) old-old adults, a total of 72 older participants (14.6% of all the older participants), had experienced falls within a year. Figure 4 shows SOT performance for fallers and non-fallers. Two-way ANOVA showed that the non-faller had greater AH than that of the faller group, although EH did not significantly differ for the faller and non-faller groups. Furthermore, 20 out of 72 fallers (27.8%) and 68 out of 422 (16.1%) non-fallers failed to step over the bar at the EH (i.e., overestimation), with these percentage data significantly differing for fallers and non-fallers (*p* < 0.05).

## Discussion

Our results revealed that 17.8% of community-dwelling older adults failed to step over the bar at the estimated maximum height (i.e., EH), whereas all the young adults succeeded in the SOT trials at EH. Furthermore, comparisons between EH, AH, and the resultant estimation error (Δ height) showed that the older adults tended to overestimate, or underestimate to a lesser extent, SOT ability, compared with the young adults. Such an overestimation in older adults has also been observed in other tasks such as reaching tasks [18,19]. Furthermore, among the older adults in the present study, overestimation was more frequent in the fallers than the non-fallers. This suggests that overestimation, or decreased underestimation, in older adults may increase the risk of falls.

### Decreased underestimation, or overestimation, in older adults and a lack of awareness of age-related decline in physical ability

Overestimation, or decreased underestimation, of SOT ability in older adults may result from a lack of awareness of age-related decline in SOT ability. This was evident in the following results: (i) AH decreased significantly as age increased, whereas EH was almost identical among the 3 age groups (Figure 2), and (ii) AH was negatively correlated with age (r = -0.454), whereas EH was correlated with age (r = -0.117) to a lesser extent in the older, but not the young, adults (Figures 3b and 3c). The unchanged EH with age implies that the older adults were not aware of their age-related decline in SOT ability as shown in AH. Such a lack of awareness of age-related decline in SOT ability, rather than decreased SOT ability per se, might lead to overestimation, or decreased underestimation, of SOT ability in older adults.

Furthermore, the self-estimation error, or Δ height (in which a positive value indicates overestimation and a negative value underestimation), appeared to be highly correlated with EH (r = 0.716) and moderately correlated with AH (r = -0.389) in the older adults, whereas in the young adults, Δ height was highly correlated with EH (r = 0.755) alone but not correlated with AH (r = -0.038). This suggests that the older adults who showed relatively low SOT ability (AH) tended to overestimate (or underestimate to a lesser extent than that of the young adults) their SOT ability, whereas this was not the case for the young adults. For the benefit of self-protection against accidents such as a fall, older adults, particularly those with poor physical ability, should underestimate rather than overestimate their physical abilities so as to perform a motor action safely [26,27]. Nevertheless, our results showed that the older adults with lower SOT ability tended to overestimate or underestimate to a lesser extent their poor physical ability, whereas the young adults showed more underestimation. Therefore, older adults, particularly those with poor physical ability, may not realize their age-related decline in physical ability, which may result in overestimation or decreased underestimation of SOT ability, as seen in reaching action [18,19].

### Decreased underestimation in older adults: Is it accuracy or inaccuracy of self-estimation?

Regarding the explanations [18,25,28] for decreased underestimation (or overestimation) in older adults, Konczak et al. [25] have suggested that decreased underestimation in older adults reflects increased accuracy of self-estimation, and that older adults may be aware of their age-related physical decline and are therefore sensitive to environmental changes in order to avoid potential accidents. In contrast, young adults have good physical ability and can thus lower the potential risk of accidents such as a fall, even if their self-estimation is not accurate (increased underestimation). Konczak et al. thus assumed that self-estimation in older adults was accurate, resulting in less underestimation than young adults, who generally show more underestimation.

Contrary to the explanation of Konczak et al. [25], our correlation analyses (Figure 3a) showed that the correlation of EH with AH was much stronger in the young adults (r = 0.624) than in the older adults (r = 0.342). This implies that the young adults consistently estimated their actual SOT ability with a high ratio of contribution (i.e., strong regression) between EH and AH, whereas the older adults estimated their actual SOT ability inconsistently/inaccurately. Therefore, the explanation of Konczak et al. for decreased underestimation in older adults may not be correct, because self-estimation in the older adults did not consistently/accurately reflect actual SOT ability, compared with the young adults.

We suggest that self-estimation in older adults inconsistently reflects actual physical ability and is influenced by certain factors, such as past physical ability, which leads to overestimation or decreased underestimation. Our results showed that estimation error (Δ height) in the older adults increased (i.e., approached overestimation) as their physical ability diminished. A possible explanation is that older adults with poor physical ability might avoid going out or participating in physical activities, and such a life style of inactivity limits recognition and re-estimation of current physical ability, with a resultant tendency to estimate physical ability according to that in their youth [29-31]. This results in overestimation, or decreased underestimation, in older adults [32,33].

Other likely explanations could relate to lower memory as well as likely age-related changes in visual perception. Previous findings [34] have suggested that lower working memory might be associated with significant overestimation of reaching ability. Although in this study we screened out older adults with low cognitive functions with a criterion of MMSE < 27, our results showed overestimation, or lesser underestimation, of the SOT ability in the older adults. Therefore, some executive functions other than those indicated by MMSE may have played an important role in producing overestimation, or lesser underestimation, of physical ability in older adults.

Furthermore, age-related changes in visual perception (e.g., height/depth perception) may also influence inaccurate self-estimation of motor ability [35,36]. However, the participants in our study had normal binocular sight. It is also likely that wearing multifocal glasses could predispose older adults to tripping or falling compared with single distance glasses [37]. Unfortunately, we did not collect any data regarding the number of older adults who worn multifocal glasses in this study. Even if wearing multifocal glasses affected visual perception, our findings in the present study cannot adequately be explained in terms of wearing multifocal glasses, because it is far from clear that how the likely age-related changes in visual perception due to wearing multifocal glasses can cause overestimation rather than underestimation which was shown in the present study. The likely effects of wearing multifocal glasses on self-estimation should then be examined in further investigations.

Regarding underestimation in young adults, Robinovitch and Cronin [18] have speculated that young adults may unconsciously ensure the safety of their motor actions and thus largely underestimate physical ability. Such underestimation would contribute a large safety margin in performing a reaching action safely while standing [18,28]. Although this conservative estimation in young adults might be explained by psychological and/or perceptional factors, this should be more closely examined in future studies.

### Decreased underestimation, or overestimation, in older adults correlating to a risk of falls

In our study, 14.6% of all the older participants reported that they had fallen in the past year. This is a relatively small number of incidence of falls in older adults compared to those in a large number of epidemiological studies [1-3,38,39]. The reason for a small number of fall experiences evident in our older participants may be that older participants tend to underreport their fall experience in retrospective reports. However, our older participants appeared both physically and cognitively healthy, with an MMSE larger than 27 (without limitations for IADL). Furthermore, our older participants were relatively young (young-older adults represented approximately 70% of the older participants). Therefore, this may explain the small number of fall experiences in our older participants.

Our comparisons between the fallers and non-fallers revealed that the percentage of participants who failed to step over the bar at EH (i.e., overestimation) was significantly larger in the fallers (27.8%) than the non-fallers (16.1%), and that actual SOT ability (i.e., AH) was significantly lower in the fallers than the non-fallers, whereas self-estimation (i.e., EH) was almost the same for both groups. Successful SOT actions require sufficient physical abilities, such as muscular strength, balance, and flexibility, that enable the performer to stand stable on a single leg with appropriate subsequent control of both legs when performing the step-over action [7,40,41]. The significantly low AH of the fallers compared with that of the non-fallers might be a crucial factor in a failure in the SOT task. More importantly, the fallers may not have correctly perceived, or recognized, their current declined physical ability (i.e., the low AH), and thus demonstrated an EH similar to that of the non-fallers. These features of AH and EH in the fallers caused decreased underestimation, or overestimation, of SOT ability, leading to a narrow safety margin for performing the SOT actions. This resultant narrow safety margin in the SOT action could be a risk for falls; this may therefore relate to the past experience of falls of the fallers.

A potential confounding factor affecting the self-estimation of step-over ability may be fear of falling. Increased fear of falling should much underestimate their step-over ability compared with non-fearful older adults. The fear of falling is strongly associated with fall experience [42,43]. However, our results showed that the older adults generally less underestimated or overestimated their SOT ability and that the percentage of fallers who overestimated SOT ability was almost double larger than that of the non-fallers. Therefore, the fear of falling could not well explain our results.

### Contribution of decreased underestimation, or overestimation, to a risk of falls differing for the step-over task and reaching task

The discrepancy between our results from the step-over task and those of previous study [19] using reaching tasks could be explained by differences in motor patterns inherent in the tasks. As referred in the introduction section, several recent studies [18,19] showed typical overestimation or decreased underestimation in reaching tasks, although it was reported that this did not relate to the occurrence of falls. In contrast, our results showed clear relationships between overestimation or decreased underestimation of SOT capability and the experience of falls. The step-over action would present a potential risk of tripping while stepping over an obstacle, whereas the reaching action per se may result in unstable balance when standing but may not directly lead to tripping. Therefore, the contribution of overestimation or decreased underestimation (i.e., a small safety margin) of task capability to a possible risk of falls might differ between step-over and reaching actions, although the occurrence of overestimation or decreased underestimation of task-related physical ability may be general irrespective of motor patterns, such as step-over action and reaching action. In examining such a different nature of step-over and reaching actions for a risk factor of falling, further longitudinal studies will be needed to elucidate relevant factors, such as cognitive function, visual perception, and psychological states, that influence a risk of falling for both step-over and reaching tasks.

## Conclusion

Our results clearly showed that older adults tended to overestimate, or underestimate to a lesser extent, physical ability which was potentially diminished with age, and that older adults with poor step-over performance tended to overestimate their ability more than older adults with good step-over performance. This may have resulted from a lack of awareness of age-related physical decline. Diminished physical ability and, more importantly, overestimation or decreased underestimation caused by unawareness of reduced physical ability may act synergistically to increase the risk of falls in older adults. This suggests that correct self-estimation, particularly avoidance of overestimation, of age-related physical decline may well be crucial in preventing falls.

## Competing interests

The authors declare that they have no competing interests.

## Authors' contributions

RS structured the study design, performed statistical analyses, interpreted data, and drafted the manuscript. KI supervised the overall processes; participated in the study design, statistical analyses, and interpretation of data; and helped finalize the manuscript. YF participated in designing this study, acquiring data, and structuring the data set. MI, TH, and HU contributed to data interpretation, suggested appropriate data analyses, and prepared the manuscript. All authors have read and approved the final manuscript.

## Pre-publication history

The pre-publication history for this paper can be accessed here:

http://www.biomedcentral.com/1471-2318/13/44/prepub
